# On the Use of Blisters in Checking the Progress of Mortification

**Published:** 1807-11

**Authors:** P. S. Physick


### *On the Use of Blisters in checking the Progress of Mortification.* By P. S. Physick, M. D.

[ Extracted from the Philadelphia Medical Museum. ]

THE practice of curing erysipelatous inflammation by the application of a blister over the inflamed part, originated, ated, as far as I know, with the late Dr. Joseph Pfeiffer. From having employed blisters in the treatment of that complaint with great success, I was induced to suppose, some years ago, that they might also be used with advantage in arresting the progress of mortification.

The first opportunity I had of applying a blister with this intention, was in the case of Captain Stokes, a gentleman between forty and fifty years of age, whom I was desired to visit, in consultation with Dr. Rush, in January 1803. After an inflammation about the anus, which had been supposed for several days by the patient an attack of piles, a mortification was observed to have commenced in the perineum, and on the side of the scrotum. At my first visit I proposed the application of a blister, to extend from the edge of the mortification in the perineum, backwards over the buttocks. This being agreed to, was immediately applied. The following day, when the blister was dressed, we were both well satisfied with its effect, as it had prevented the mortification from spreading backwards; but so extensive was the mortification of the skin and anterior part of the scrotum, which appeared to extend upwards in the course of the spermatic chords towards the abdomen, that his recovery was not to be expected. After a few days he died.

Dr. Rush being struck with the good effect of the blister in the preceding instance, has lately employed the remedy in a case of mortification, the history of which is contained in the following letter.

"Dear Sir,

"I was called upon by Dr. Bleight, on the 29th of last July, to visit with him, Captain R. A. who, in consequence of applying a handful of the *polyconum persicaria,* instead of paper, to a common use, after going to stool, was affected with an inflammation in the extremity of the rectum, which extended around the adjoining parts, and along the perineum, so as to affect the integuments of the scrotum. Bleeding and other depleting remedies had been used to no purpose, in order to cure it: a partial mortification had taken place. I concurred with Dr. Bleight in advising leeches to the sound parts; and recollecting the high terms in which you spoke of the efficacy of blisters in preventing the progress of mortifications, in our consultation, in the case of Captain Stokes, in January 1803, I advised their application to all the diseased parts which had not put on a gangrenous appearance. They had the wishedwished-for effect; the mortified parts were afterwards cut away, or gradually sloughed off; and, under the faithful and patient subsequent attendance of Dr. Bleight, the Captain happily recovered, and now enjoys his usual health.

"In the most dangerous state of his disease, we gave him bark; but its distressing effects upon his system obliged us to lay it aside.

"From, dear Sir,

"Your sincere Friend,

"BENJAMIN RUSH."

DR. P. S. PHYSICK,
*Nov.* 15*th*, 1804.

On the 24th October 1804, I was desired to meet Doctors S. P. Griffitts, Wistar, and Strattan, in consultation, concerning the case of Mr. Charles French, who was afflicted with a mortification of the foot, which was advancing daily upwards, unchecked by the liberal use of the bark. On the 27th October, I proposed the application of a large blister round the leg, below the knee; this being agreed to, was applied in the evening; when dressed the next morning, it was observed that the mortification had not increased:—encouraged by the benefit derived from it, I proposed on the 29th, the application of a second blister, to cover all the living parts below the edge of the first. This blister also rose well;—in a few days a distinct line of separation between the living and dead parts was observed; the blisters were dressed with a mixture of basilicon and spirit of turpentine. I avoid relating further particulars of this case, as Dr. Griffitts proposes to publish a circumstantial detail of it.

Since writing the above, I have been favoured with the following history of a case from Dr. Church, containing additional testimony in favour of the use of blisters in arresting the progress of gangrene.

"ON Monday, ——— of November, I was desired to visit Mrs. Y. in the country, about sixty years of age, of a fair complexion, and delicate constitution; has had several children, and heretofore enjoyed good health.

"She had been taken on Saturday with frequent chills, with irregular febrile flushings, pain in the limbs and head, which continued increasing for nearly thirty-six hours before I saw her, when she was delirious, with flushed countenance, irregularly frequent and intense pulse, tongue furred, respiration frequent, with great general uneasiness.

"The

"The loss of ten or twelve ounces of blood, with a saline cathartic, abated in some degree the febrile symptoms. The delirium still continuing, in the evening, blisters to the wrists, with a continuation of the saline mixture, produced an alleviation of all the symptoms, so that towards morning she had a few hours sleep. When she awoke, she was perfectly collected, complaining of great soreness in her body and limbs, particularly in one ankle, which she said was painful; her skin was cool, and her pulse frequent but soft, easily yielding to the least pressure. The family informed me that the ankle she complained of, had had an ulcer on it for fourteen years, which had been brought on by a slight injury after one of her deliveries; that it had within the last two weeks been healed. On examination, the ulcer appeared to have been of the size of a dollar, above the internal ankle, which was now quite livid, with some swelling around the edges, having the appearance of a vesication, and a deep purple blush, extending an inch or two beyond it, attended with a distressing burning sensation. The leg at this time was quite cool and somewhat swelled.

"The medicines she had been taking were omitted, and the tonic cordial plan substituted: the bark, wine, and opium were administered freely, and cataplasms of bark with yeast, were applied to the part, and changed frequently.

"This treatment was followed until Thursday, with an increase of the lividity and vesications on different parts of the ankle, filled with a bloody-coloured fluid.

"On Thursday the appearances were indeed unpleasant; the lividity of the ankle had extended, and the deep purple colour of the skin was near the middle of the leg, with very great tumefaction. The pulse was frequent, skin cool, tongue dry, and much apparent insensibility of the limb. The bark was still continued internally, and the fermenting cataplasm of powdered carbone, with meal, honey, and yeast, was applied in large quantities over the part affected, and was repeated or changed very frequently. This plan was originally adhered to all Thursday and Friday, changing the bark (which now had been taken in such quantities as to sicken the stomach) for some other tonic.

"The deep and burning redness, still, however, progressed towards the knee, with an increase of those unpleasant vesications; the pulse on Saturday was much more frequent; the skin cool; tongue dry, and covered with with a dark-coloured crust; very great restlessness, with constant incoherent muttering.

"In this situation I recollected a conversation I had had some time since with Dr. Physick, in which he mentioned the good effects he had experienced from blistering, in a case of gangrene. The critical and dangerous state of the patient required something to be promptly done. The blister was proposed with considerable hesitation, as I could not recollect in what stage or what species of gangrene Dr. Physick had used it.

"A large blister was, however, applied on the inside of the leg below the knee, one part on the healthy portion of the leg, and the remainder immediately on the diseased part. After twelve hours it rose very well, and, contrary to what I dreaded, assumed a very pleasing aspect, and without the least increase of disease. The pulse still continued frequent and the skin cool, although the patient in every other respect was much more composed.

"On Sunday, the leg was much more favourable; the lividity and vesication had not increased, and the tumefaction, which was very considerable, had subsided much. The foot, though much swelled before, did not, until this period, shew the least disposition to take on diseased action. It now became covered with the deep purple shining appearance, with a distressing burning sensation, which, together with the increased tumefaction, occasioned much uneasiness. The bark, with elixir of vitriol, was persevered in freely, and from the pleasing effects of the blister, in arresting the rapid progress of the disease in the leg, I applied a large one, covering all the upper part of the foot, including that part of the ankle where the disease first began. The effects were equally as pleasing as in the first instance, producing an almost immediate cessation of the progress of the disease.

"The parts of the ulcer where the disease first began, separated to some depth; the cuticle from below the knee separated, and in some places on the leg; and the separation extended even through the cutis and adipose membrane.

"The tumefaction of the leg gradually diminished, and the patient is completely free from every danger."

Impressed with an idea that blisters will be often found useful in preventing the progress of mortifications, I have been induced to publish the preceding cases as early as possible.

*Philadelphia, 24th November,* 1804.

